# New drugs for the control and elimination of malaria: a snapshot of the pipeline

**DOI:** 10.1186/1475-2875-11-S1-O31

**Published:** 2012-10-15

**Authors:** Timothy NC Wells

**Affiliations:** 1 MMV, Geneva, Switzerland

## 

Medicines are having a terrific impact on the lives of malaria patients. Great progress has been made with understanding the safety and efficacy of fixed dose artemisinin combination therapies, with five medicines either prequalified by WHO or soon to be prequalified, allowing treatment of children for as little as 25 cents. Artesunate injections are now being established as standard of care for severe malaria, offering significant improvement in healthcare, at a low cost from prequalified suppliers. Medicines can also be used to protect vulnerable populations. Seasonal malaria chemoprophylaxis can protect children for less than 60 cents per year; chemoprophylaxis in pregnancy has significant potential for the lives of the mothers and babies. However, we are facing an infectious enemy, which can and does develop resistance. New medicines are now in phase II clinical trials which have the potential to overcome any emerging resistance, and offer the hope for a single dose cure. We have programs to develop new safer molecules to block transmission of the parasite and to prevent relapses, and these are starting to define potential clinical candidates, after four years of investment. Finally, the need for chemoprotection means that we need new medicines with high potency and long duration. To facilitate these needs have released a ‘malaria box’: a set of 400 physical compounds, and also all the data on over 20’000 hits. Over the next few years, then innovative new partnerships with industry and academia based on this open access to data will help us define a new era in antimalarial drug development.

